# P04 Temocillin for OPAT treatment of urinary tract infections: A single-centre case series

**DOI:** 10.1093/jacamr/dlaf239.008

**Published:** 2026-01-05

**Authors:** Ben McIntyre, Rohit Bazaz, Kirsty Dodgson, Michael Riste

**Affiliations:** Manchester University NHS Foundation Trust, Manchester, M13 9WL, UK; Manchester University NHS Foundation Trust, Manchester, M13 9WL, UK; Manchester University NHS Foundation Trust, Manchester, M13 9WL, UK; Manchester University NHS Foundation Trust, Manchester, M13 9WL, UK

## Abstract

**Background:**

MDR Enterobacterales are an increasingly common cause of urinary tract infections (UTIs) and present significant treatment challenges. These pathogens are usually resistant to most, and sometimes all, oral antibiotic options and hospital admission for IV therapy with carbapenems is often necessary. Temocillin is a narrow-spectrum agent with stability against many ESBLs and AmpC enzymes. In April 2020, EUCAST published clinical breakpoints for certain Enterobacterales species and categorized those with MIC ≤16 mg/L as I (susceptible, increased exposure). This further recommended temocillin only be used for infections of urinary origin and in practice would necessitate 2 g three times daily dosing.

**Objectives:**

We aimed to evaluate the use, appropriateness and clinical outcomes of temocillin at standard doses (up to 2 g twice daily) for OPAT treatment of UTI.

**Methods:**

We conducted a retrospective review of all adult patients treated for UTI with temocillin via the OPAT service at a large NHS Trust (Manchester University NHS Foundation Trust) over a 12 month period between 1 April 2024 and 31 March 2025. The OPAT service maintains a comprehensive patient database of all referrals, which was used to identify patients eligible for inclusion and search electronic health records. Baseline demographics, UTI diagnosis, microbiological data, treatment duration and clinical outcomes were documented and analysed. Significant adverse events and 60 day hospital re-admission or re-referral to OPAT for UTI were also recorded. Outcomes were categorized according to UK good practice recommendations for OPAT (2019).

**Results:**

A total of 27 OPAT episodes were included, representing 24 individual patients (female, 16; 58%). Median age was 70 years (range 26–88). Twenty-one cases (78%) had a diagnosis of complicated UTI (including pyelonephritis, urosepsis and catheter-associated infection). Gram-negative organisms in urine and/or blood cultures were present in 25 cases (93%) prior to starting treatment. Concurrent bacteraemia was present in 11 cases (44%). ESBL/AmpC-producing organisms accounted for 22 (88%) of all positive cultures (*Escherichia coli* [10]; *Klebsiella pneumoniae* [6]; *Morganella morganii* [5]; *Proteus mirabilis* [1]). The remainder were either non-ESBL/AmpC *E. coli* or mixed growth/no predominant organism. Standard dosing of temocillin was used for all patients, with doses ranging from 1 g od to 2 g bd and adjusted for renal function where appropriate. Median total duration of therapy was 8 days (range 3–14). Treatment aim (cure) was achieved in all patients, with a single episode of acute kidney injury and hyperkalaemia as the only recorded adverse event. Three OPAT episodes (11%) resulted in re-presentation to Trust services with UTI symptoms within 60 days of discharge from OPAT. There was one patient death of unknown causes.

**Conclusions:**

Our experience supports the use of temocillin at standard dosing up to 2g twice daily for OPAT treatment of Gram-negative urinary tract infections and is a particularly valuable agent for those caused by resistant organisms. In our study cohort, temocillin achieved excellent clinical outcomes and was shown to be very well tolerated. Temocillin also has important antimicrobial stewardship advantages as a narrow-spectrum, carbapenem-sparing agent where once-daily ertapenem may otherwise be the only alternative OPAT option.

